# High-grade appendiceal mucinous neoplasm presenting as a giant appendiceal mucocele

**DOI:** 10.1016/j.radcr.2021.02.014

**Published:** 2021-02-24

**Authors:** Alan Lu, Junsang Cho, Maryna Vazmitzel, Lester Layfield, Kevin Staveley-O'Carroll, Ayman Gaballah, Deepthi Rao

**Affiliations:** a University of Missouri – Columbia School of Medicine, Columbia, MO; b Department of Pathology, University of Missouri Health Care, Columbia, MO, USA 65212; c Department of Surgery, University of Missouri Health Care, Columbia, MO; d Department of Radiology, University of Missouri Health Care, Columbia, MO

**Keywords:** Appendiceal mucinous neoplasm, High grade, Low grade, Pseudomyxoma peritonei, Appendiceal mucocele

## Abstract

Appendiceal mucinous neoplasms are rare findings defined by an accumulation of mucus within the vermiform appendix, and can be caused by a variety of conditions. Appendiceal mucinous neoplasms are important to consider because they can develop into pseudomyxoma peritonei as a consequence of perforation. We report a case of a 55-year-old man who initially presented with increasing abdominal girth, constipation, anorexia, and unintentional weight loss. Computed tomography examination of the abdomen and pelvis demonstrated a huge thin-walled cystic mass causing significant displacement of the surrounding abdominal and pelvic structures. The mass was amenable to resection and removed without perforation. Gross pathologic examination demonstrated a 44.0 × 40.0 × 23.0 cm unilocular cystic mass with a section of attached bowel. Microscopic examination revealed high-grade appendiceal mucinous neoplasm arising in a background of low-grade appendiceal mucinous neoplasm. This case report provides an evidence to include appendiceal mucinous neoplasms in the differential diagnosis of large abdominal cystic masses.

## Introduction

Appendiceal mucinous neoplasm (AMN) is one of the causes of appendiceal mucocele, which is marked by dilation of the appendix from mucus accumulation [1]. Mucoceles most commonly present as acute appendicitis; however, they can also present with gastrointestinal (GI) bleeding, intussusception, and obstruction. These entities have historically been discovered incidentally [2]. If a mucocele caused by an appendiceal mucinous neoplasm ruptures, the patient is at risk of developing pseudomyxoma peritonei (PMP) - a clinical condition marked by mucus accumulation in the peritoneum leading to progressive abdominal disease and intestinal obstruction [3]. For this reason in addition to challenges with cytologic diagnosis, biopsy of mucinous appendiceal lesions is contraindicated. Physicians must also have a high degree of suspicion for appendiceal mucinous neoplasms when treating abdominal cystic masses, and care should be taken during surgical removal of mucoceles to avoid disruption of the mass. If an appendiceal mucinous lesion is confined to the appendix, appendectomy is performed for definitive pathologic diagnosis.

In this report, we describe a case of a 44.0 cm mucocele caused by a high-grade appendiceal mucinous neoplasm (HAMN), which is significantly larger than any other previously reported case. In addition, we present the histological features that led to the diagnosis, discuss the classification of appendiceal mucinous neoplasms, as well as treatment options and prognosis.

## Case report

**Fig. 1:**
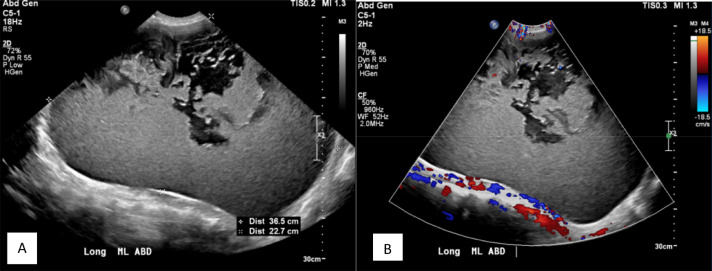
Grey scale (A) and color Doppler (B) longitudinal ultrasound images of the abdomen and pelvis showing a huge mixed echogenicity mass measuring up to 36.5 cm. The mass is predominantly hyperechoic with irregular hypoechoic areas anteriorly and regular outline. Color Doppler image showed no significant flow within the mass, confirming the cystic nature of the mass.

**Fig. 2:**
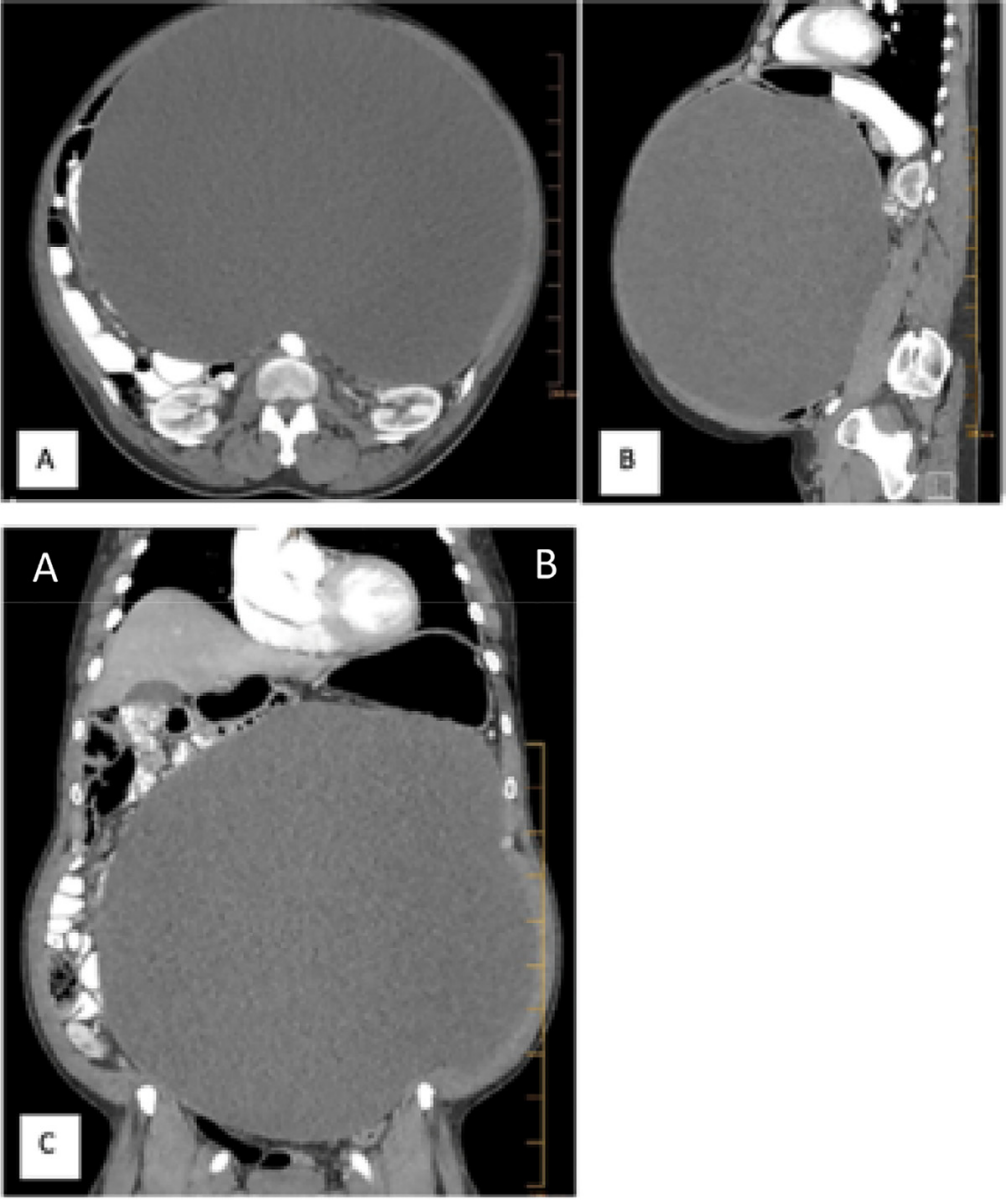
Contrast-enhanced axial (A), sagittal (B), and coronal (C) CT images showing a huge low attenuation abdominopelvic with attenuation value of 16-25 HU. No solid enhancing components or calcifications within the mass. The lesion displaces the surrounding structures and causes contour bulge of the abdominal wall.

A 55 year-old man with medical history significant for hypertension presented to his primary care physician (PCP) with complaints of progressively increasing abdominal girth of 1 year duration, accompanied by unintentional weight loss, constipation, and poor appetite. On physical exam, the patient's height was 166.8 cm and weight was 86.2 kg. The patient's abdomen was distended, diffusely tender and firm, and measured 49 inches in circumference. Abdominopelvic ultrasound and contrast-enhanced computed tomography (CT) scan were performed. Ultrasound examination showed a huge mixed echogenic mass measuring 36.5 cm within the central portions of the abdomen and pelvis (Fig. 1). No significant intra lesional color Doppler flow was noted (Fig. 1B). Due to the large size of the mass, it was difficult to assess its entire extent or relations to the surrounding structures. Cross-sectional imaging was recommended for further evaluation. Contrast-enhanced CT of the abdomen and pelvis showed a 36.0 × 25.0 × 33.0 cm lobulated thin-walled cystic mass with thin internal septations. The attenuation value of the mass ranged from 16 to 25 Hounsfield units. No associated enhancing components or calcifications were noted within the lesion. There was significant mass effect with displacement of the surrounding abdominal and pelvic structures (Fig. 2). No masses were detected within the solid abdominal organs. The biliary tree and main pancreatic duct were normal in caliber. The CT scan and ultrasound results provided a broad differential considerations including mesenteric cyst, lymphangioma, enteric duplication cyst, or mesothelial cyst. Upon review of the imaging results, it was decided that the mass would be amenable to resection.

**Fig. 3:**
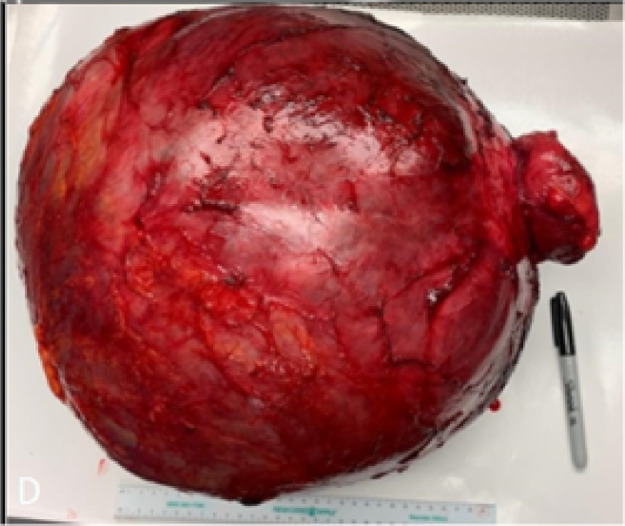
Gross image of 18.6 kg, 44 × 40 × 23 cm mass representing an intact, unoriented pink-red unilocular cystic mass with a small portion of attached bowel.

At surgery, the mass was well-circumscribed, with adhesions to the abdominal wall and the bowels. It strongly adhered to the lower right colon, near the cecum. There was a palpable otomy between the cecum and the mass. The mass was resected entirely intact while maintaining the ileocecal valve. No visible vermiform appendix was seen during the operation. The mass was sent to pathology for intraoperative consult. It was received as an intact, unoriented pink-red unilocular cystic mass with a small portion of attached bowel. The specimen weighed 18.6 kg, and cystic mass measured 44.0 × 40.0 × 23.0 cm (Fig. 3). The mass was opened and revealed approximately 18.0 kg of dark-red mucinous and bloody fluid. The inner lining was smooth and glistening without papillary excrescences. The mass did not grossly appear to communicate with the portion of attached bowel. A section of the wall was taken for a frozen section, which showed a mucinous cystic neoplasm.

**Fig. 4:**
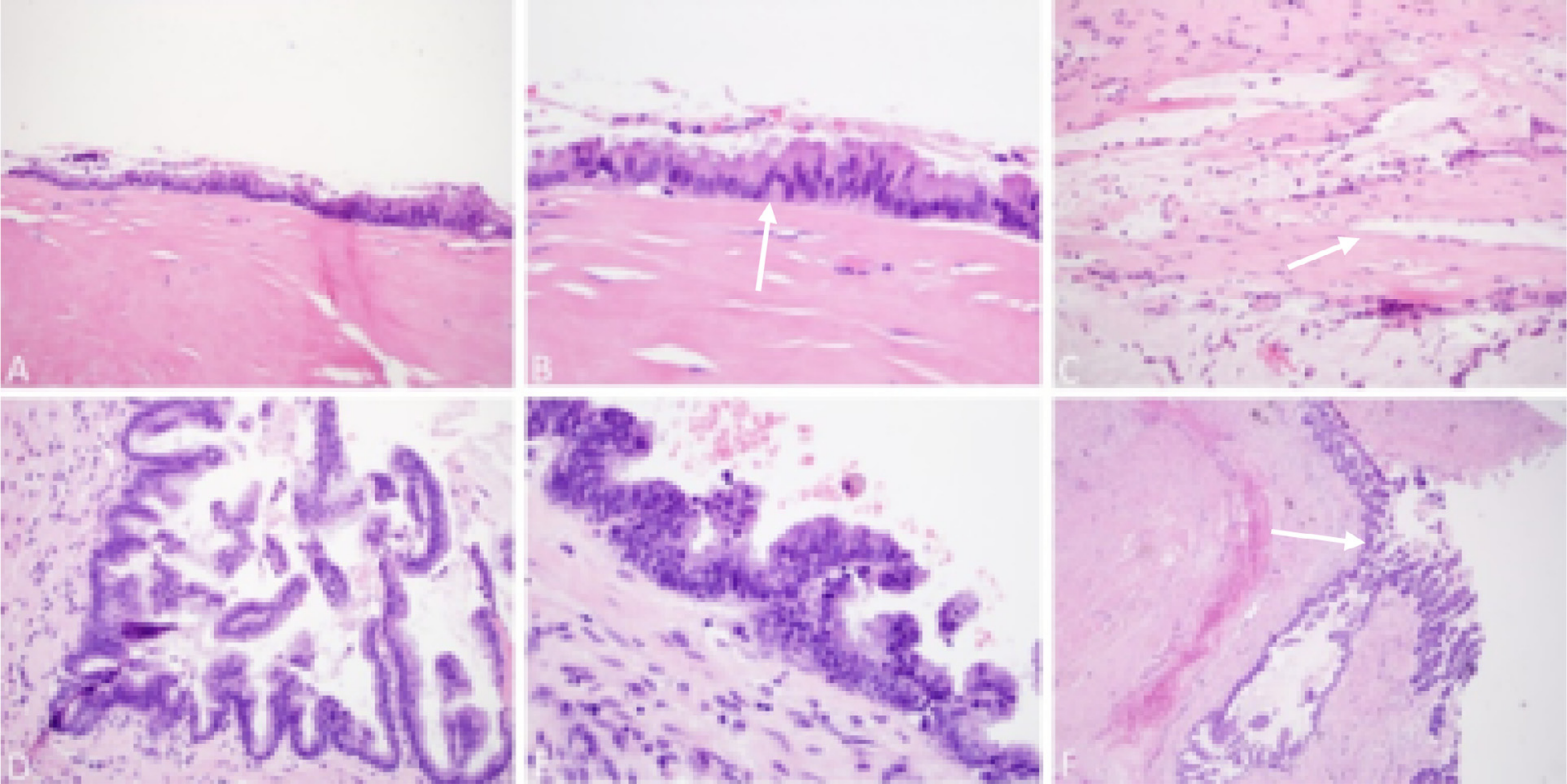
Hematoxylin and eosin (H&E) stain (A) Monolayer of mucinous epithelium, with hyalinization of wall and loss of muscularis mucosa and submucosa; (B) Mucinous epithelium with low-grade cytologic dysplasia; (C) Dissection of mucin in wall of mass; (D) Focal complex architectural changes; (E): High-grade cytologic features; (F): Pushing margin into wall of appendix.

Additional sections of the mass were taken for permanent sections. The wall of the mass showed diffuse hyalinization, with loss of muscularis mucosa and submucosa (Fig. 4A). The majority of the mass showed replacement of mucosa by a single layer of mucinous epithelium with low-grade cytologic dysplasia. The nuclei were basally oriented, relatively uniform, and appeared elongated and hyperchromatic, with no significant mitotic activity seen (Fig. 4B). The wall also showed dissection of mucin in focal areas (Fig. 4C). A few foci demonstrated areas of complex architectural changes with nuclear psuedostratification, micropapillary structures, and presence of focal cribriform pattern (Fig. 4D). In addition, there was high-grade dysplasia, loss of polarity, and markedly enlarged pleomorphic and hyperchromatic nuclei (Fig. 2. E). One area with high-grade dysplasia demonstrated a pushing margin into the wall of the appendix (Fig. 2. F). These features led to the diagnosis of HAMN, which was arising in a background of low-grade appendiceal mucinous neoplasm (LAMN).

Following the surgery, the patient did well. He initially required 2 units of packed red blood cells for anemia and experienced postoperative ileus. After some time, the patient gradually advanced to a regular diet and was able to ambulate in the hallways without trouble. On the thirteenth postoperative day, the patient tolerated a regular diet and exhibited bowel movements. He was subsequently discharged and is still doing well to date.

## Discussion

Appendiceal mucinous neoplasms are a heterogeneous group of neoplasms ranging from simple mucoceles to complex pseudomyxoma peritonei. Mucoceles are subset that consists of gradual cystic dilation of the vermiform appendix due to slow accumulation of mucoid contents. It is uncommonly seen, occurring in between 0.2% and 0.4% of appendectomies and 0.3 %and 0.7% of all appendiceal pathology [1]. There are several classes of conditions that may cause an appendiceal mucinous neoplasm. These include simple mucoceles (with degenerating epithelium causing obstruction), hyperplastic mucoceles (due to hyperplasia of the appendix and cecal mucosa), and also neoplasms. Simple and hyperplastic mucoceles comprise up to 25% of cases, with neoplastic etiologies comprising the remainder [4]. Ultrasound and CT are valuable imaging modalities for detecting AMN and are often utilized in emergency settings [8]. Characteristic ultrasound findings include a mass with fine echo spots and/or concentric, echogenic layers (also known as “onion skin”) [5]. On CT scan, characteristic features include cystic dilation of the appendix with low attenuation, mural calcification, and a luminal diameter greater than 1.3 cm.

The differential considerations of an abdominopelvic cystic lesion are wide and include ruptured appendicitis, mucinous cystadenoma, lymphoma, cecal carcinoma, ovarian and fallopian tube pathology that may mimic smaller appendiceal mucinous neoplasms. Characteristic findings for ruptured appendicitis on CT scan include significant periappendiceal inflammatory changes, focal defect in the enhancing appendiceal wall, extraluminal gas, or extraluminal leak of enteric contrast [6]. Mucinous cystadenoma, whether from the appendix, ovary, or pancreas, is larger than a serous cystadenoma and may exhibit mural calcifications. In addition, mucinous cystadenomas on MRI are usually seen as large multilocular cystic lesions containing fluid of various viscosity. Variable signal intensities on both T1 and T2 sequences can be seen according to the mucin content [7]. In general, the cell type (eg, serous vs mucinous) and severity (eg, adenoma, carcinoma) often cannot be determined on the basis of imaging appearance. However, the presence of enhancing nodular components and thick septations should raise the possibility of malignant transformation [7]. Excision is necessary to determine the exact diagnosis. Preoperative diagnosis can be aided through the assistance of imaging modalities which will prevent complications including PMP. However, the definitive diagnosis of an appendiceal mucinous neoplasm vs the aforementioned differential on imaging is made intraoperatively and on histopathological examination.

The classification of mucinous neoplasms of the appendix is controversial and different terminologies have been used to describe these lesions. Recent efforts to build a consensus naming system have led to the development of a classification system that includes LAMN, HAMN, and mucinous adenocarcinomas [8,9,22]. The term “mucinous adenocarcinoma” is generally reserved for mucinous lesions with features of infiltration into the appendiceal wall, while LAMN and HAMN refer to noninvasive lesions with varying degrees of cytologic atypia.

Clinically, LAMN presents most often as acute appendicitis, and is more common in females, and in the sixth decade of life. Appendices that have LAMN can either be dilated by mucin, or can appear unremarkable [10]. The typical microscopic appearance of a LAMN is a mucinous epithelium that is villous or flat in architecture [11]. Extensive collection of mucin can lead to increased pressure that causes atrophy and attenuation of the mucinous lining [12]. This epithelium overlies hyalinized stroma, which has replaced the normal lamina propria, muscularis mucosa, and submucosa. This hyalinization is one sign of the pushing-border invasion that helps define this lesion [11]. Other histologic features that can be observed are dissection of acellular mucin into the wall, and rupture of the appendix with mucin and epithelial cells seen outside of the appendix [8]. There are several differential diagnoses to consider in diagnosing LAMN, including reactive changes in epithelium, mucinous adenoma, and invasive mucinous carcinoma [12]. Targeted next-generation sequencing of LAMNs has shown that KRAS and GNAS mutations are common [13,14].

HAMN is a relatively new term, resulting from a consensus of the Peritoneal Surface Oncology Group International in 2015. Previously, this entity was referred to as “noninvasive mucinous adenocarcinoma” or “cystadenocarcinoma,” which was deemed to be unclear and inconsistent with other terminology for appendiceal neoplasms [9]. Microscopically, this lesion has similar changes in the appendiceal wall as LAMN, showing pushing-border invasion into the wall with no infiltration. However, the cytologic atypia seen in the epithelium is of higher grade than what is seen in LAMN. Based on mutational analysis and morphologic evidence, LAMN and HAMN are thought to have a common histogenesis, and it is likely that HAMN arises from LAMN. The 2 entities share high rates of mutations in KRAS and GNAS, and HAMNs often harbor additional TP53 and ATM mutations [13,14]. For this reason, in cases of LAMN, it is important to submit the appendix specimen entirely for microscopic examination, to ensure that there is no focus of HAMN or invasive carcinoma.

A literature search for LAMN, HAMN, and appendiceal mucocele showed that the largest reported case of appendiceal mucinous cystic neoplasm measured approximately 17 cm in greatest dimension [15], [16], [17], [18], [19], [20]. The neoplasm that we are reporting measures 44 cm in greatest dimension, which is significantly larger than any other previously reported case. In addition, our diagnosis of HAMN is uncommon and clinically poorly understood due to its rarity. Including HAMN in the differential diagnosis for abdominal cystic masses is critical because mucinous neoplasms are known to be the most common causes of PMP - a potentially lethal condition marked by deposition of pools of mucin in the peritoneum. Treatment for patients with PMP may involve extensive de-bulking in combination with intraoperative hyperthermic intraperitoneal chemotherapy [21]. Rupture of a mucinous neoplasm during surgery puts the patient at further increased risk of dissemination into the peritoneum and causing PMP. For noninvasive appendiceal mucinous neoplasms, wide mesoappendiceal resection is recommended to evaluate for lymph node spread [21]. In addition, right hemicolectomy should be considered for positive margins, tumor size of 2 cm or greater, or tumors exhibiting high grade histology. Invasive mucinous adenocarcinoma may also require extensive resection in the case of lymph node positivity [23]. Recent research has also provided evidence that neoadjuvant chemotherapy consisting of FOLFOX (Folinic acid, Fluorouracil, and Oxaliplatin combination therapy) before receiving serial debulking and hyperthermic intraperitoneal chemotherapy therapy may be effective in metastatic disease or AMN perforation [24].

In conclusion, we report a case of HAMN, which is the larger than any other previously reported. HAMN and other mucinous neoplasms of the appendix must be included in the differential diagnosis for all cystic abdominal masses because rupture of such lesions can lead to PMP. Special efforts must be made during surgery to avoid rupture of cystic masses, especially appendiceal mucinous neoplasms. Further research and trials will have to be performed to outline standardized procedures in treating AMN including performing debulking surgery, chemotherapy use, and medical management for patients who are not surgical candidates. In addition, for patients with large neoplasms, standard of therapy is not outlined and may require physician judgment on anatomic structures that will have to be removed. Pathologists and surgeons will need to continue gathering data to identify a standardized diagnostic and treatment plan for AMNs in the future.

## Patient consent statement

Patient was notified and consented for the publication of this report.
